# Depression and anxiety symptom networks across the lifespan

**DOI:** 10.1093/ageing/afaf153

**Published:** 2025-06-06

**Authors:** Daniel Harlev, Aya Vituri, Moni Shahar, Noham Wolpe

**Affiliations:** Department of Physical Therapy, Stanley Steyer School of Health Professions, Gray Faculty of Medical & Health Sciences, Tel Aviv University, Tel Aviv 6997801, Israel; Rambam Health Care Campus, Department of Psychiatry, Haifa 31999, Israel; Tel Aviv Center for Artificial Intelligence & Data Science (TAD), Tel Aviv University, 6997801, Israel; Tel Aviv Center for Artificial Intelligence & Data Science (TAD), Tel Aviv University, 6997801, Israel; Department of Physical Therapy, Stanley Steyer School of Health Professions, Gray Faculty of Medical & Health Sciences, Tel Aviv University, Tel Aviv 6997801, Israel; Sagol School of Neuroscience, Tel Aviv University, Tel Aviv 6997801, Israel

**Keywords:** centrality, depression, anxiety, network analysis, ageing, older people

## Abstract

**Background:**

The relationship between anxiety and depressive symptoms is complex and may vary across the lifespan. Symptom network analyses offer a powerful tool to examine these interactions, but few studies have directly compared symptom networks in younger and older adults.

**Methods:**

We analysed data from the Cambridge Centre for Ageing and Neuroscience (Cam-CAN) study, including 786 participants aged 18 to 88, who reported at least subclinical levels of symptoms on the Hospital Anxiety and Depression Scale (HADS). Network analysis was employed to examine symptom communities (clusters of related symptoms), within- and between-community connectivity (association strength), and centrality (symptom importance) across age groups.

**Results:**

The overall network structure, separating anxiety and depressive symptoms into two communities, remained stable. However, older adults showed reduced connectivity within depression and between depression and anxiety. While ‘panic’ was a consistently central symptom, ‘rumination’ and ‘restlessness’ were the key bridge symptoms (i.e. linking anxiety and depression) in young and older adults, respectively.

**Discussion:**

Our findings reveal both stable and dynamic aspects of depression and anxiety symptoms across the lifespan. Reduced within-community connectivity for depressive symptoms suggests greater heterogeneity in how depression manifests in older populations. The shift in bridging symptoms, from cognitive (rumination) in young adults to somatic (restlessness) in older adults, suggests subtle yet clinically important differences in how depression and anxiety are linked across the lifespan. Our findings support age-informed assessment and diagnosis of depressive and anxiety symptoms.

## Key Points

- Depression and anxiety symptom networks evolve with age, with stable and dynamic aspects.
- Older adults show reduced within-community connectivity for depressive symptoms.
- Bridging symptoms shift from cognitive (rumination) in young adults to somatic (restlessness) in older adults.
- Between-community connectivity declines with age, reducing depression-anxiety interactions.
- Findings highlight the need for tailored, age-dependent interventions.

## Introduction

Depression is a common and disabling condition, marked by the heterogeneity of its symptoms and their severity [1, 2]. The impact of even mild ‘subclinical’ depressive symptoms on well-being [3, 4], and the substantial overlap and frequent co-occurrence of depressive and anxiety symptoms [5], challenge the traditional categorical view of these conditions as distinct entities. Instead, dimensional models may better reflect the complexity and overlap of symptoms [6].

Network analysis offers a powerful tool for examining symptom relationships within this dimensional framework [7, 8]. By visualising and quantifying connections between symptoms, researchers and clinicians can identify central symptoms (‘hubs’) that may be crucial for understanding and treating these disorders. In this framework, individual symptoms are represented as nodes, and statistical associations between them as edges, whose strength (connectivity) can be quantified. Importantly, measures such as centrality—reflecting how strongly a symptom is connected to others—and bridge centrality—indicating how strongly a symptom links different symptom communities (e.g. anxiety and depression)—may provide insights into mechanisms that sustain psychopathology [8]. Network analysis can thus reveal critical insights into the dynamics of depression and anxiety symptoms, potentially informing clinical strategies by highlighting influential symptoms that sustain or connect broader symptom domains [9].

A significant contributor to symptom heterogeneity is age. The clinical presentation of symptoms of depression and anxiety differs between younger and older adults [10, 11]. While older adults tend to report more somatic symptoms, young adults predominantly report mood and interpersonal symptoms [12]. Network methods can capture these age-related differences in symptom structure.

Prior studies analysed symptom networks separately by age group. These studies have yielded mixed findings regarding the relationship between depression and anxiety symptoms, with inconsistencies in network structures and symptom centrality patterns within groups [8, 13–16]. For example, ‘worthlessness’ and ‘loss of interest’ were central in younger adults [8], while ‘uncontrollable worry’ and ‘trouble relaxing’ were central in older adults [15, 16]. To our knowledge, no study has directly compared the symptom networks of young and older adults.

This study aims to address this gap by directly comparing symptom networks in young and older adults, utilising data from the general population across the lifespan through the Cambridge Centre for Ageing and Neuroscience (Cam-CAN) study. We employed symptom network analysis to examine the structure and centrality of depressive and anxiety symptoms. By using a large, population-based sample and a standardised assessment tool, we aimed to provide a robust comparison of symptom networks across the lifespan. We hypothesised similar community structures across age, but with reduced connectivity and centrality in older adults, and different age-specific central symptoms.

## Methods

### Participants

We analysed data from the Cam-CAN, a population-based study conducted in Cambridgeshire, UK, between 2010 and 2016 [17]. Participants were recruited through referrals from general practitioners. Of 7616 eligible individuals, 2681 completed Stage 1, and 2598 provided complete data on all 14 HADS items and were included in the current analyses (response rate ~34%). Ethical approval was obtained from the Cambridgeshire 2 Research Ethics Committee (reference: 10/H0308/50), and all participants provided written informed consent. We defined ‘young adults’ as individuals aged 18–45 years and ‘older adults’ as those aged 65+ years.

### Measures and scales

Participants completed demographic questionnaires and the Hospital Anxiety and Depression Scale (HADS), which includes 14 items scored 0–3 [18]. We included individuals scoring a total of ≥4 on the HADS-D subscale, indicating at least subclinical depressive symptoms [3].

### Network computation and analysis of key metrics

We computed symptom networks for the 14 HADS items (seven anxiety and seven depression symptoms). Each item was treated as a node, and connections (edges) between items were computed using mutual information (MI), which is a non-parametric statistic capturing both linear and non-linear dependencies [19]. Communities (i.e. symptom clusters) were identified using the Louvain algorithm, which maximises modularity [20]. The procedure is fully data-driven and requires no prior labelling. As a validation step, we repeated all analyses using symptom clusters as defined by HADS (anxiety vs. depression items).

Within- and between-community connectivity were operationalised as the mean edge weight among symptoms within or across communities, respectively. To compare connectivity values between age groups and communities, we used non-parametric permutation tests (5000 iterations). We also computed two centrality metrics: strength centrality (total connectivity per symptom) and bridge strength (connectivity to symptoms in other communities). Only strength-based metrics were used, consistent with recommendations for analyses of fully connected graphs. Spearman correlations were used to compare centrality rankings across age groups. Further methodological details are provided in the Supplementary Data.

## Results

Our cohort comprised 786 participants (232 young adults and 554 older adults). Key demographic and clinical characteristics, including age, sex, education, socioeconomic status, alcohol intake and antidepressant use, are summarised in Table 1. Internal consistency for the HADS in our study was acceptable and comparable to previous studies (*α* = 0.79 for HADS-A, α = 0.72 for HADS-D) [18]. No significant group differences were found for overall depressive symptom severity (*t*(408.20) = 0.71, *P* = .475), whereas overall anxiety symptom severity was significantly higher (*t*(433.07) = 9.16, *P* < .001) in younger adults (*M* = 8.25, *SD* = 3.56) compared to older adults (*M* = 5.70, *SD* = 3.55).

**Table 1:** Demographic and clinical characteristics of the study sample by age group (young vs. older adults). Alcohol use was coded as follows: 1 = daily or almost daily, 2 = three or four times a week, 3 = once or twice a week, 4 = one to three times a month, 5 = special occasions only, 6 = never. Education was coded according to the English education system: 1 = college or university degree or higher, 2 = a levels/AS levels or equivalent, 3 = O levels/GCSEs or equivalent, 4 = CSEs or equivalent. Psychotropic medication includes the use of at least one antidepressant or anxiolytic medication.

Variable	Young	Old	Statistic	*P*-value
Age	33.42 ± 7.05	81.29 ± 7.10		
Sex	107/232	223/554	*χ* ^2^ = 0.73,	*P* = .392
HADS-D	6.32 ± 2.47	6.19 ± 2.31	*t* = 0.71,	*P* = .475
HADS-A	8.25 ± 3.56	5.70 ± 3.55	*t* = 9.16	*P* < .001
Education	2.73 ± 0.67	1.76 ± 1.41	*U* = 85418.00 *P* < .001	*P* < .001
Alcohol use	3.65 ± 1.41	3.52 ± 1.88	*U* = 63187.50	*P* = .556
Psychotropic medication use	25/232	80/554	*χ* ^2^ = 2.40,	*P* = .121

Before turning to the network analyses, we first examined the severity of each depressive and anxiety symptom across age groups (Table 2), by conducting pairwise comparisons for each item and correcting for multiple comparisons using the false discovery rate (FDR) procedure [21]. Younger adults generally reported significantly higher severity in most anxiety symptoms, while differences in depressive symptoms were more mixed. Older adults reported higher severity in specific depressive symptoms related to fatigue (HD4), decreased motivation (HD6) and sustained enjoyment (HD1), while younger adults reported higher severity in symptoms related to depressed mood (HD3) and anhedonia (HD7). However, our focus was on the structure of the relationship between these symptoms, for which we employed a symptom network approach.

**Table 2:** Depressive and anxiety item comparison between younger and older adults. The table presents the mean scores (±SD) for each HADS item by age group. Values represent self-rated symptom severity on a 4-point Likert scale ranging from 0 (not at all) to 3 (most of the time), as per HADS scoring. *P*-values were obtained from independent *t*-tests comparing the means between the two groups and were corrected for multiple comparisons using the FDR procedure.

Item	Young Mean (SD)	Old Mean (SD)	*U*-Statistics	*P*-value (FDR Corrected)
Enjoyment (HD1)	0.46 (0.67)	0.61 (0.78)	415579.0	<0.001
Laughter (HD2)	0.26 (0.50)	0.27 (0.54)	466113.0	1.00
Cheerful feeling (HD3)	0.35 (0.54)	0.27 (0.52)	500249.0	0.001
Slowed down (HD4)	0.70 (0.72)	1.36 (0.93)	282006.0	<0.001
Loss of interest in appearance (HD5)	0.50 (0.74)	0.47 (0.71)	471670.0	1.00
Look forward to things (HD6)	0.37 (0.65)	0.53 (0.72)	408737.5	<0.001
Enjoyment of book/media (HD7)	0.28 (0.63)	0.19 (0.52)	492970.0	0.004
Feeling of tension (HA1)	1.06 (0.65)	0.78 (0.62)	566009.5	<0.001
Frightened feeling (HA2)	0.69 (0.81)	0.53 (0.74)	513962.0	<0.001
Worrying thoughts (HA3)	0.98 (0.87)	0.76 (0.80)	531450.0	<0.001
Relaxed feeling (HA4)	0.84 (0.68)	0.60 (0.63)	551580.0	<0.001
Butterflies in stomach (HA5)	0.61 (0.64)	0.43 (0.59)	538413.0	<0.001
Restless feeling (HA6)	1.21 (0.90)	0.92 (0.82)	547494.5	<0.001
Feeling of panic (HA7)	0.54 (0.67)	0.49 (0.65)	483132.0	0.362

### Depression-anxiety symptom networks and communities

**Figure 1:**
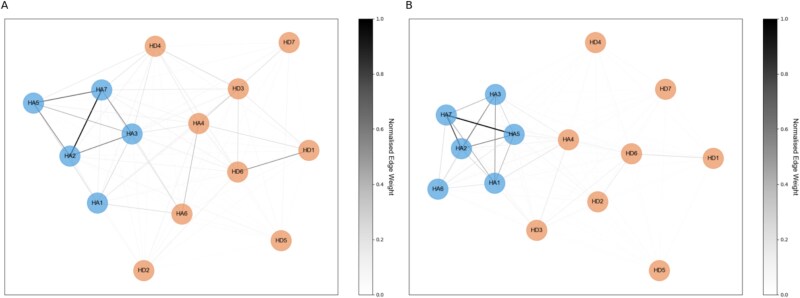
Depression-anxiety symptom networks in younger and older adults. (A) Network constructed using MI between HADS questionnaire items in younger adults, with edges representing the magnitude of MI associations between items. Edge thickness and colour (as indicated in the colour bar) indicate normalised MI. Node colours represent communities detected by the Louvain algorithm. (Blue shade: anxiety community, orange shade: depression community). (B) Same as (A) but for older adults.

After computing depression-anxiety symptom network for each age group, we conducted a community detection analysis to identify distinct clusters or communities of symptoms within the networks (Figure 1). In the network of younger adults, two communities were detected. One community predominantly included anxiety-related items (HA1-HA3, HA5, HA7), while the other community included depressive items (HD1-HD7) along with the anxiety items ‘relaxed feeling’ (HA4) and ‘restless feeling’ (HA6). In older adults, the network showed a similar community structure. There were again two communities, with a small overlap between depressive and anxiety items. One community predominantly included anxiety-related items (HA1-HA3 and HA5-HA7), while the other community included depression-related items (HD1-HD7) and the anxiety item ‘relaxed feeling’ (HA4). This suggests that symptom communities remained largely similar across age groups, with two predominant depression- and anxiety-related symptom communities.

We analysed the network based on these communities, which were detected in a data-driven manner. However, to confirm that any observed group differences in these analyses are not due to the small differences in symptom clustering into communities between age groups, we also performed these analyses on the symptom clusters according to their assignment in the HADS, i.e. one community for anxiety items (HA) and another community for depression items (HD). The results were similar to those described below (see Supplementary Results and Fig. S1 in Supplementary Data).

### Within- and between-community connectivity

**Figure 2:**
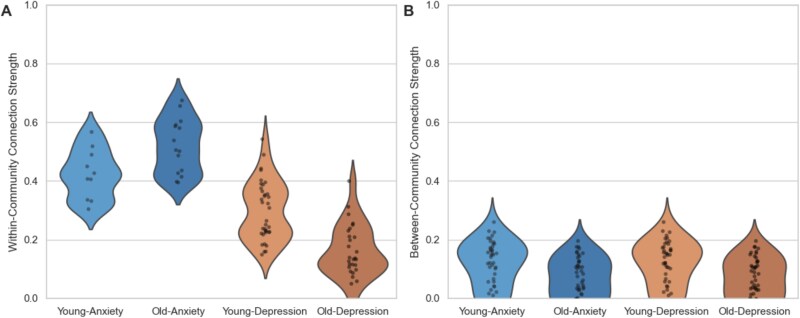
Age-related differences in within- and between-community connectivity. (A) Violin plots displaying the distribution of within-community connection strength for each symptom within the depression (orange shade) and anxiety (blue shade) symptom communities, for both young (lighter shades) and older adults (darker shade). (B) Same as (A) but for between-community connections.

We examined both within-community connectivity (strength of connections within each community) and between-community connectivity (strength of connections between communities) for both age groups (Figure 2). Overall, the anxiety-related symptom community exhibited higher within-community connectivity compared to the depression-related symptom community across both age groups. Importantly, there was an age-related increase in within-community connectivity specifically for the anxiety-related symptom community, with significantly higher connectivity in older adults (*M* = 0.519) compared to young adults (*M* = 0.423; *P* < .001, permutation test). In contrast, within-community connectivity in the depression-related symptom community significantly decreased with age, showing lower connectivity in older adults (*M* = 0.160) compared to young adults (*M* = 0.298; *P* < .001, permutation test).

For between-community connections, across both communities, we found a significant age-related decline in between-community connectivity (*P* < .001, permutation test) in older adults (*M* = 0.0625) compared to young adults (*M* = 0.1112). This pattern was consistent for both the depression and anxiety communities.

### Strength centrality and bridge strength

**Figure 3:**
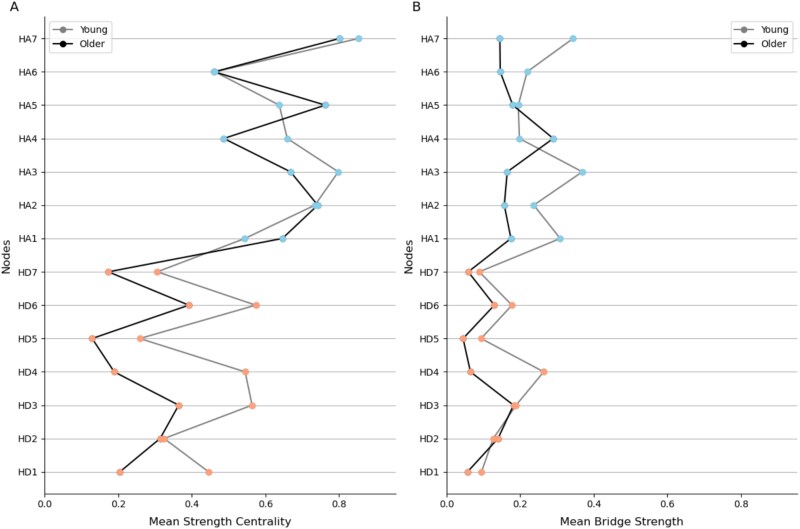
Comparison of centrality measures between age groups. (A) Strength centrality, representing the sum of connection weights for each symptom, is displayed for both young (grey line) and older adults (black line). Nodes are coloured according to their classification in the HADS, with anxiety symptoms (HA) in blue and depressive symptoms (HD) in orange. (B) Bridge strength centrality, which quantifies the importance of a symptom in connecting the depression and anxiety communities, is shown for young and older adults. Each node is represented by a grey line connecting its bridge strength values in the young (grey line) and older adults (black line) groups. Node colours correspond to their classification in the HADS, as in (A).

We examined both strength centrality and bridge strength measures across age groups. In terms of strength centrality (Figure 3A), item HA7 (‘panic’) exhibited the highest strength centrality in both young and older adult groups. Moreover, across items, strength centrality pattern appeared similar between age groups. A rank-based correlation analysis showed a near-perfect alignment in symptom centrality ranking between age groups (*ρ* = 0.999, *P* < .001), suggesting that the influence of individual symptoms within the whole network was largely stable with age.

In contrast to the consistent yet reduced centrality measures for the depressive symptom community, bridge strength pattern differed with age (Figure 3B). Specifically, HA3 (‘rumination’) exhibited the highest bridge strength in young adults, whereas HA4 (‘restlessness’) exhibited the highest bridge strength in older adults. Spearman correlation analysis showed a moderate positive correlation between the rank of bridge strength scores in young and older adults (*ρ* = 0.51, *P* = .064), indicating a weak consistency in the bridging role of nodes across age groups.

For completeness, we report a set of exploratory analyses on middle-aged subgroup (individuals aged 46–64, *n* = 187) to explore age as a continuum. The network showed a similar structure to that of the young group, with two communities and matching item composition. The most central nodes by strength were HA3 (‘rumination’) and HA7 (‘panic’). HA3 also showed the highest bridge strength (see Supplementary Results and Figs. S2 and S3 for full details).

## Discussion

Our study employed network analyses to examine the complex structure of depressive and anxiety symptoms in the general population across the lifespan. Our principal findings include an overall stability with age for network organisation and centrality; an age-related increase in within-community connection strength for anxiety but decrease in within-community connection for depression; age-specific bridging symptoms, with ‘rumination’ and ‘restlessness’ as the most prominent bridging symptoms in young and older adults, respectively. Together, the results suggest that anxiety and depression symptom networks evolve with age, which has important implications for age-specific tailoring of treatment.

Direct comparisons of symptom severity showed age-related differences. Younger individuals with subclinical depressive symptoms tended to report more severe anxiety symptoms compared to older adults. This observation aligns with previous research highlighting a stronger association between depression and anxiety in younger populations [22]. However, some studies have reported contrasting findings, potentially due to age-related biases in assessment tools or the influence of other age-dependent factors, such as physical health comorbidities, cognitive decline, social isolation and bereavement [23, 24].

In terms of symptom networks, symptom communities remained consistent across age groups, and largely mapped onto depressive and anxiety symptoms of the HADS. This suggests that the core organisation of psychopathological networks might be preserved across different populations and contexts [25].

Interestingly, some anxiety symptoms were clustered into the depressive symptom community and vice versa for some depressive symptoms. Specifically, ‘difficulties in relaxation’ and ‘restless feelings’ were clustered into the depression-related community. This suggests a potential blurring of the boundaries between depression and anxiety, particularly in relation to somatic symptoms. Moreover, previous studies using the HADS have found a three-dimensional model, which includes a ‘psychomotor agitation’ dimension that is strongly correlated with depression [26, 27]. While our network analysis did not explicitly identify three distinct communities, the closer association of certain anxiety symptoms with the depressive community suggests a potential overlap between these dimensions.

Examining the connections within and between communities, a notable result was the age-related decline in the connectivity of the depressive symptom community, while connectivity in the anxiety community significantly increased with age. This divergence suggests age-specific changes in how these symptom domains interact and manifest. The decline in depressive symptom connectivity may reflect greater heterogeneity in reported depressive symptoms among older adults, potentially driven by comorbid medical conditions and social isolation [28, 29]. From a clinical perspective, this age-related change might imply not only that depressive symptoms are less likely to cluster together in older adults, but also that targeting a single or core symptom might be less effective in alleviating the broader depressive experience in this age group. Evidence for smaller clinical efficacy of depression treatment in older adults is widely reported [10, 30], calling for more individualised treatment approaches. In contrast, the increased within-community connectivity of anxiety symptoms suggests a more consolidated anxiety presentation in older adults, which could reflect heightened interrelatedness or a tendency for these symptoms to co-occur more strongly with age [31].

Between-community connectivity declined significantly with age, suggesting a diminishing interplay between depression and anxiety symptoms. This reduction was particularly pronounced for connections involving anxiety symptoms. This finding might reflect age-related changes in cognitive-emotional processing, including shifts in attention, memory and emotional regulation, which can alter the interplay between affective symptoms [32, 33]. Knight and Durbin [34] further propose that older adults may develop more adaptive coping mechanisms, such as prioritising emotionally meaningful goals and reducing engagement in ruminative thinking, which may contribute to a reduced coupling of depression and anxiety symptoms. As cognitive control processes change with age, particularly in the context of emotion regulation and thought patterns, the distinction between depression and anxiety may become more pronounced. Future studies should investigate how cognitive changes, particularly in executive functions and emotional regulation, contribute to these age-related alterations in symptom connectivity and how these insights could guide age-specific therapeutic strategies.

The analysis of symptom centrality again revealed some age-related consistencies but also discrepancies in the structure of depression-anxiety symptom network. In both age groups, anxiety symptoms, and particularly ‘panic’, were more central within the symptom network compared to depressive symptoms [8], echoing some who suggest anxiety symptoms play a prominent role in the overall experience of distress relative to depressive symptoms [35]. Importantly, we observed an age-related difference in bridging symptoms, with rumination emerging as the strongest bridge symptom in younger adults, but restlessness emerging as the strongest bridge symptom in older adults. Bridging symptoms are particularly informative, as they reflect symptoms that statistically link distinct symptom domains—in this case, depression and anxiety—and may therefore serve as potential conduits for comorbidity [36]. For instance, in younger adults, individuals experiencing persistent ruminative thoughts may be more likely to also report anxiety symptoms, whereas in older adults, somatic agitation such as restlessness may similarly link anxiety and depressive symptoms. These findings suggest a potential shift in the mechanisms underlying comorbidity, from cognitive-affective pathways in youth to more somatic manifestations in older age. Supporting this interpretation, evidence suggests that depression and anxiety can manifest differently in older adults, with a greater emphasis on somatic complaints and physical symptoms [31, 37]. Clinically, this highlights the need to tailor screening and diagnostic tools to the symptom profiles most relevant for each age group in order to facilitate the identification of those at risk for mixed depressive-anxiety presentations.

### Strengths and limitations

Our study has several key strengths. We used validated scales (HADS) within a large, population-based cohort encompassing a wide age range. Participants were more representative of the general population than in many other ageing cohorts, due to sampling through primary care in the UK, and were balanced in terms of sociodemographic characteristics, with a similar number of individuals in each age decile [17]. Networks were computed using MI, which is a robust statistical method for detecting linear and non-linear relationships between variables.

However, our study also has several limitations. First, the cross-sectional design precludes causal inference or the assessment of symptom changes over time. Second, the reliance on self-report measures rather than clinical interviews introduces a potential bias. Third, although our focus on a non-clinical population offers insights into real-world symptom patterns, it may limit generalisability to diagnosed clinical populations. Fourth, the HADS does not capture the full range of affective symptoms defined in diagnostic manuals. Fifth, the study’s reliance on voluntary participation introduces potential selection bias, as individuals with more severe depressive or anxiety symptoms may have been less likely to take part. Sixth, the Cam-CAN cohort was originally designed to study cognitive ageing, which limits the depth of available affective symptom data. Seventh, while we included descriptive comparisons of demographic and clinical variables across age groups, our network models did not adjust for confounders, as can be done in linear models. Finally, while our main analyses compared young and older adults, this approach does not capture the full developmental trajectory. A supplementary analysis of middle-aged adults (aged 46–64 years) suggested a broadly similar network to that of younger adults, though not identical. Longitudinal studies are needed to examine how symptom networks evolve with age and to identify potential transitional patterns.

## Conclusion

We found evidence for both stability and age-related differences in anxiety-depression symptom network. The core network structure and the centrality of certain symptoms remained consistent within a group. However, in older compared to young adults, differences emerged specifically for depressive symptoms, where there was a transition from cognitive to somatic bridging symptoms, and a decline in within-community connectivity. These results suggest that while anxiety symptoms maintain their relationship with age, depressive symptoms are more heterogeneous, which may suggest different underlying mechanisms. These findings underscore the need for tailored clinical approaches that recognise both the consistent and evolving nature of depression and anxiety symptoms across the lifespan. Future research would further investigate the mechanisms underlying these age-related differences and their implications for treatment.

## Supplementary Material

aa_25_0266_File002_NW_DH_afaf153

## References

[1] Buch AM, Liston C. Dissecting diagnostic heterogeneity in depression by integrating neuroimaging and genetics. Neuropsychopharmacology 2021;46:156–75. 10.1038/s41386-020-00789-3. 32781460 PMC7688954

[2] Gutiérrez-Rojas L, Porras-Segovia A, Dunne H et al. Prevalence and correlates of major depressive disorder: a systematic review. Braz J Psychiatry 2020;42:657–72. 10.1590/1516-4446-2020-0650. 32756809 PMC7678895

[3] Cuijpers P, Smit F. Subclinical depression: a clinically relevant condition? Tijdschr Voor Psychiatr 2008;50:519–28. 18688776

[4] Noyes BK, Munoz DP, Khalid-Khan S et al. Is subthreshold depression in adolescence clinically relevant? J Affect Disord 2022;309:123–30. 10.1016/j.jad.2022.04.067. 35429521

[5] Zbozinek TD, Rose RD, Wolitzky-Taylor KB et al. Diagnostic overlap of generalized anxiety disorder and major depressive disorder in a primary care sample. Depress Anxiety 2012;29:1065–71. 10.1002/da.22026. 23184657 PMC3629816

[6] Vink D, Aartsen MJ, Schoevers RA. Risk factors for anxiety and depression in the elderly: A review. J Affect Disord 2008;106:29–44. 10.1016/j.jad.2007.06.005. 17707515

[7] Fried EI, Epskamp S, Nesse RM et al. What are “good” depression symptoms? Comparing the centrality of DSM and non-DSM symptoms of depression in a network analysis. J Affect Disord 2016;189:314–20. 10.1016/j.jad.2015.09.005. 26458184

[8] Park S-C, Kim D. The centrality of depression and anxiety symptoms in major depressive disorder determined using a network analysis. J Affect Disord 2020;271:19–26. 10.1016/j.jad.2020.03.078. 32312693

[9] van Borkulo C, Boschloo L, Borsboom D et al. Association of Symptom Network Structure with the course of depression. JAMA Psychiatry 2015;72:1219–26. 10.1001/jamapsychiatry.2015.2079. 26561400

[10] Alexopoulos GS . Mechanisms and treatment of late-life depression. Transl Psychiatry 2019;9:188. 10.1038/s41398-019-0514-6. 31383842 PMC6683149

[11] Bergmann E, Harlev D, Cam-CAN et al. Depressive symptoms are linked to age-specific neuroanatomical and cognitive variations. Journal of Affective Disorders. 2025;369:1013–20. 10.1016/j.jad.2024.10.077. 39442700

[12] Schaakxs R, Comijs HC, Lamers F et al. Age-related variability in the presentation of symptoms of major depressive disorder. Psychol Med 2017;47:543–52. 10.1017/S0033291716002579. 27786143

[13] Beard C, Millner AJ, Forgeard MJC et al. Network analysis of depression and anxiety symptom relationships in a psychiatric sample. Psychol Med 2016;46:3359–69. 10.1017/S0033291716002300. 27623748 PMC5430082

[14] Kaiser T, Herzog P, Voderholzer U et al. Unraveling the comorbidity of depression and anxiety in a large inpatient sample: network analysis to examine bridge symptoms. Depress Anxiety 2021;38:307–17. 10.1002/da.23136. 33465284

[15] Yang T, Guo Z, Cao X et al. Network analysis of anxiety and depression in the functionally impaired elderly. Front Public Health 2022;10:1067646. 10.3389/fpubh.2022.1067646. 36530716 PMC9751796

[16] Zhang P, Wang L, Zhou Q et al. A network analysis of anxiety and depression symptoms in Chinese disabled elderly. J Affect Disord 2023;333:535–42. 10.1016/j.jad.2023.04.065. 37086797

[17] Shafto MA, Tyler LK, Dixon M et al. The Cambridge Centre for Ageing and Neuroscience (Cam-CAN) study protocol: a cross-sectional, lifespan, multidisciplinary examination of healthy cognitive ageing. BMC Neurol 2014;14:204–25. 10.1186/s12883-014-0204-1. 25412575 PMC4219118

[18] Bjelland I, Dahl AA, Haug TT et al. The validity of the hospital anxiety and depression scale: an updated literature review. J Psychosom Res 2002;52:69–77. 10.1016/S0022-3999(01)00296-3. 11832252

[19] Wolpe N, Vituri A, Jones PB et al. The longitudinal structure of negative symptoms in treatment resistant schizophrenia. Compr Psychiatry 2024;128:152440. 10.1016/j.comppsych.2023.152440. 38039918

[20] Dugué N, Perez A . Directed Louvain: Maximizing Modularity in Directed Networks. Orléans, France: Université d’Orléans; 2015. Available from: https://hal.science/hal-01231784.

[21] Benjamini Y, Yekutieli D. The control of the false discovery rate in multiple testing under dependency. Ann Stat 2001;29:1165–88. 10.1214/aos/1013699998.

[22] Beekman ATF, de Beurs E, van Balkom AJLM et al. Anxiety and depression in later life: co-occurrence and communality of risk factors. Am J Psychiatry 2000;157:89–95. 10.1176/ajp.157.1.89. 10618018

[23] Jorm AF . Does old age reduce the risk of anxiety and depression? A review of epidemiological studies across the adult life span. Psychol Med 2000;30:11–22. 10.1017/S0033291799001452. 10722172

[24] Wuthrich VM, Johnco CJ, Wetherell JL. Differences in anxiety and depression symptoms: comparison between older and younger clinical samples. Int Psychogeriatr 2015;27:1523–32. 10.1017/S1041610215000526. 25892278

[25] Boschloo L, Van BCD, Rhemtulla M et al. The network structure of symptoms of the diagnostic and statistical manual of mental disorders. PloS One 2015;10:e0137621. 10.1371/journal.pone.0137621. 26368008 PMC4569413

[26] Caci H, Baylé FJ, Mattei V et al. How does the hospital and anxiety and depression scale measure anxiety and depression in healthy subjects? Psychiatry Res 2003;118:89–99. 10.1016/S0165-1781(03)00044-1. 12759165

[27] Friedman S, Samuelian J-C, Lancrenon S et al. Three-dimensional structure of the hospital anxiety and depression scale in a large French primary care population suffering from major depression. Psychiatry Res 2001;104:247–57. 10.1016/S0165-1781(01)00309-2. 11728614

[28] Jellinger KA . The heterogeneity of late-life depression and its pathobiology: a brain network dysfunction disorder. J Neural Transm 2023;130:1057–76. 10.1007/s00702-023-02648-z. 37145167 PMC10162005

[29] Korten NC, Comijs HC, Lamers F et al. Early and late onset depression in young and middle aged adults: differential symptomatology, characteristics and risk factors? J Affect Disord 2012;138:259–67. 10.1016/j.jad.2012.01.042. 22370067

[30] Schaakxs R, Comijs HC, Lamers F et al. Associations between age and the course of major depressive disorder: a 2-year longitudinal cohort study. Lancet Psychiatry 2018;5:581–90. 10.1016/S2215-0366(18)30166-4. 29887519

[31] Wolitzky-Taylor KB, Castriotta N, Lenze EJ et al. Anxiety disorders in older adults: a comprehensive review. Depress Anxiety 2010;27:190–211. 10.1002/da.20653. 20099273

[32] Charles ST, Carstensen LL. Social and emotional aging. Annu Rev Psychol 2010;61:383–409. 10.1146/annurev.psych.093008.100448. 19575618 PMC3950961

[33] Mather M, Knight M. Goal-directed memory: the role of cognitive control in older adults’ emotional memory. Psychol Aging 2005;20:554–70. 10.1037/0882-7974.20.4.554. 16420131

[34] Knight BG, Durbin K. Aging and the effects of emotion on cognition: implications for psychological interventions for depression and anxiety. PsyCh J 2015;4:11–9. 10.1002/pchj.84. 26263526 PMC5889128

[35] Kessler RC, Chiu WT, Jin R et al. The epidemiology of panic attacks, panic disorder, and agoraphobia in the National Comorbidity Survey Replication. Arch Gen Psychiatry 2006;63:415–24. 10.1001/archpsyc.63.4.415. 16585471 PMC1958997

[36] Jones PJ, Ma R, McNally RJ. Bridge centrality: a network approach to understanding comorbidity. Multivar Behav Res 2021;56:353–67. 10.1080/00273171.2019.1614898. 31179765

[37] Balsis S, Cully JA. Comparing depression diagnostic symptoms across younger and older adults. Aging Ment Health 2008;12:800–6. 10.1080/13607860802428000. 19023732

