# Supplementary material for: Depression and anxiety symptom networks across the lifespan
Source: Age Ageing. 2025 Jun 6;54(6):afaf153. doi: 10.1093/ageing/afaf153 (PMC12143471; doi:10.1093/ageing/afaf153)
Supplement: aa_25_0266_File002_NW_DH_afaf153 [file aa_25_0266_file002_nw_dh_afaf153.docx]

## Depression and Anxiety Symptom Networks Across the Lifespan

## SUPPLEMENTARY DATA

**Supplementary Methods**

*Participants*

We analysed data from the Cambridge Centre for Ageing and Neuroscience (Cam-CAN), which is a comprehensive research project investigating cognition across the lifespan [1]. The study was conducted between 2010 and 2016, and participants were recruited via random sampling from primary care patient lists in Cambridgeshire, UK. In the UK, this method closely approximates a representative sample of the population, as registration with general practitioners is nearly universal. Of 7,616 eligible individuals invited to participate, 2,681 completed Stage 1 (CC3000), and 2,598 provided complete HADS data and were included in the analyses, yielding a response rate of approximately 34%. Ethical approval was granted by the local ethics committee, Cambridgeshire 2 (now East of England – Cambridge Central) Research Ethics Committee (reference: 10/H0308/50). All participants provided written informed consent prior to study enrolment. This study was conducted in accordance with the ethical principles outlined in the Declaration of Helsinki. We defined “young adults” as those aged 18–45 years and “older adults” as those aged 65 years and older.

*Measures and scales*

Participants included in the current analysis completed a Stage 1 home interview that incorporated demographic questionnaires (age, sex, education, medical history) and the Hospital Anxiety and Depression Scale (HADS) [2], which is a 14-item self-report questionnaire with depression (HADS-D) and anxiety (HADS-A) subscales. We included participants who scored 4 or above on the HADS-D subscale, indicating the presence of at least subclinical depressive symptoms [3,4]. This decision was based on evidence suggesting that even mild depressive symptoms can have significant implications for individuals' life [5,6], and is consistent with previous theoretical, data-based network structure approach [7]. Symptom severity was analysed using the full 0–3 item-level response scale for each HADS item. Internal consistency (Cronbach’s alpha) for the full sample was acceptable and consistent with previous studies: α = 0.79 for the HADS-A and α = 0.72 for the HADS-D [8].

*Network analyses*

1. **Network computation**

To compute the networks, we used similar methodology of previous network analysis studies that examined the structure of depressive and anxiety symptoms cross-sectionally [9], and other psychiatric symptoms including schizophrenia [10,11]. We constructed a network for all 14 HADS items, i.e., HA1-HA7 and HD1-HD7. HA1–HA7 and HD1–HD7 refer to the seven anxiety and seven depression items of the HADS, respectively. A full list of these items and their descriptive statistics appears in Table 2. For each item (‘node’), we computed its relationships (‘edges’) with all other 13 items. This was done separately for young and older adults. Relationship or edge strengths were calculated using the mutual information measure (MI), where the edge strength between items X and Y of the HADS was calculated by:


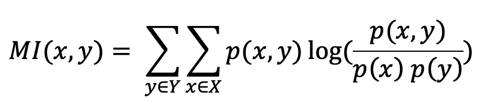


In words, MI is the sum over all possible values of X and Y of: the joint probability of X and Y, times the log of the joint probability of X and Y, divided by the product of the marginal probability of X and the marginal probability of Y. In simpler terms, mutual information captures the strength of the statistical relationship between each pair of HADS symptoms, including both linear and non-linear associations.

We retained all connections, including weaker ones, to capture the full range of symptom relationships, even those that might be weak or subtle, as even small connections can be clinically meaningful in understanding the complex interplay of symptoms [12]. The networks computed were therefore fully connected. Networks were drawn using the default Fruchterman-Reingold force-directed layout algorithm [13], in which symptoms with stronger and more numerous connections are positioned closer together and more centrally within the network. We analysed the depression-anxiety networks for the two age groups, as described below. All analyses were conducted using NetworkX package [14] in Python version 3.11.5.

1. **Community detection:** First, we used the Louvain community detection algorithm to identify clusters or communities of highly interconnected symptoms within the network [15]. Communities represent groups of symptoms that are more strongly associated with each other than with symptoms in other communities. The Louvain method finds the “optimal” number of communities in a network, which maximises modularity (highest within-community connections and lowest between-community connections). The best fit community structure is fully data-driven, and does not require any a priori constraints. We next conducted several analyses on these communities, as described below. However, to confirm that the results of these analyses were not driven by age-related differences in community clustering, we conducted similar analyses on the a priori symptom assignment of the HADS, with one community for anxiety symptoms (HA1-HA7) and another community for depressive symptoms (HD1-HD7).
2. **Within- and between-community connections:** Once communities were identified, we examined the strength of connections within- and between-community. We calculated the mean within-community connection strength as the average strength of edge weights for within-community edges and mean between-community connection strength as the average strength of edge weights for edges connecting symptoms between communities. These metrics were calculated for each community and for each age group network, and we compared them across communities and age using permutation tests, as done in previous research [16]. Specifically, in each permutation test, we randomly shuffled the age group labels (or community assignments in the case of community comparison) and recalculated the difference in mean edge weight between the two groups being compared. This process was repeated 5,000 times to generate a null distribution of differences under the assumption of no true difference between the groups. The p-value was then calculated as the proportion of permutations where the absolute difference in mean edge weight was equal to, or greater than, the observed absolute difference in the original data.
3. **Strength centrality and bridge centrality:** We calculated metrics of strength centrality and bridge centrality for each symptom across the network. First, centrality measures reflect how strongly connected a particular symptom is to all other symptoms across the whole network. We focused on node strength centrality, a widely-used and reliable metric defined as the sum of all connections a given symptom has with other nodes [17]. Strength centrality is known to align well with other centrality metrics and is often considered the most suitable indicator of symptom centrality in psychopathology networks, due to its ability to capture the overall influence of a symptom within the network [18]. Other centrality measures, such as closeness and betweenness were not computed, as they have no clear meaning for full graphs, i.e., fully connected graphs, such as those used in our study [19,20]. Second, bridge strength was calculated, which assesses the importance of a symptom in connecting different communities within the network [20]. Specifically, bridge strength for a node was calculated as the sum of the weights of its edges that connect to nodes in other communities. This differs from strength centrality, which considers connections to all nodes regardless of community membership. We used Spearman correlation analysis to examine the consistency of strength centrality and bridge strength patterns between young and older adult groups [21].
4. To complement the age-group comparison, we additionally constructed a symptom network for a middle-aged subgroup (ages 46–64) as a supplementary analysis. The network was built using same approach, and strength and bridge centrality metrics were computed for all nodes (see Fig. S2 for centrality metrics and Fig. S3 for network structure).

**Supplementary Results**

To address potential age group differences in symptom clustering, we re-ran all analyses reported in the main text using *a priori*-defined communities based on HADS symptom assignment—one community for all anxiety symptoms (HA1-HA7) and another community for all depression symptoms (HD1-HD7). This `analysis confirms that the main results were not solely driven by the small age-related differences in community clustering identified using the Louvain method (see Fig. S1 for visualization).

*Within-Community Connectivity:* We first compared the average within-community connection strength for the depression and anxiety communities across both age groups. Consistent with the results from communities detected using the Louvain method, the anxiety community exhibited higher average within-community connection strength compared to the depression community across both age groups (Young-Anxiety: *M* = 0.447, Older-Anxiety: *M* = 0.527; Young-Depression: *M* = 0.209, Older-Depression: *M* = 0.127). Significant age-related differences were observed; specifically, within-community connection strength for the depression community declined significantly with age (*p* < 0.001, permutation test), while the within-community connection strength in the anxiety community was also significantly higher in older adults compared to younger adults (*p* = 0.0018, permutation test).

*Between-Community Connectivity:* We examined between-community connectivity. As expected in the *a priori* analysis, between-community connectivity was equal for the depression and anxiety communities within each age group; however, we observed a significant age-related decline, with lower connectivity in older adults (Anxiety/Depression: *M* = 0.0625, *SD* = 0.0451) compared to young adults (Anxiety/Depression: *M* = 0.1112, *SD* = 0.0776; *p* < 0.001, permutation test).

*Consistency with Main Results:* Overall, these results align with the main findings using communities detected with the Louvain algorithm, suggesting that observed differences in connectivity patterns are not solely driven by age-related changes in community clustering. This analysis further supports that age-related differences in connectivity patterns are robust and not dependent on the method of community detection.

A supplementary network was estimated for the middle-aged subgroup (ages 46–64). Of the 612 individuals in this age range, 187 met inclusion criteria of HADS-D score ≥ 4 and complete HADS responses, and were included in the network analysis. The network structure was similar to that of the younger group, with two communities corresponding to anxiety and depression symptoms and identical item composition. The most central nodes by strength were HA3 (“rumination”; 1.04) and HA7 (“panic”; 0.98). HA3 also exhibited the highest bridge strength (0.53), indicating a central bridging role, similar to the young adult group (see Fig. S2 for centrality metrics and Fig. S3 for network structure).

*Data and Code Availability*

The code used to perform all analyses and generate figures is publicly available at: https://github.com/dharlev/depression_anxiety_network_analysis.

**Figure S1.** **A priori comparison of age-related differences in within- and between-community connectivity.** (A) Violin plots displaying the distribution of within-community connection strength for each symptom within the depression (orange shade) and anxiety (blue shade) symptom communities, for both young (lighter shades) and older adults (darker shade). (B) Same as (A) but for between-community connections.


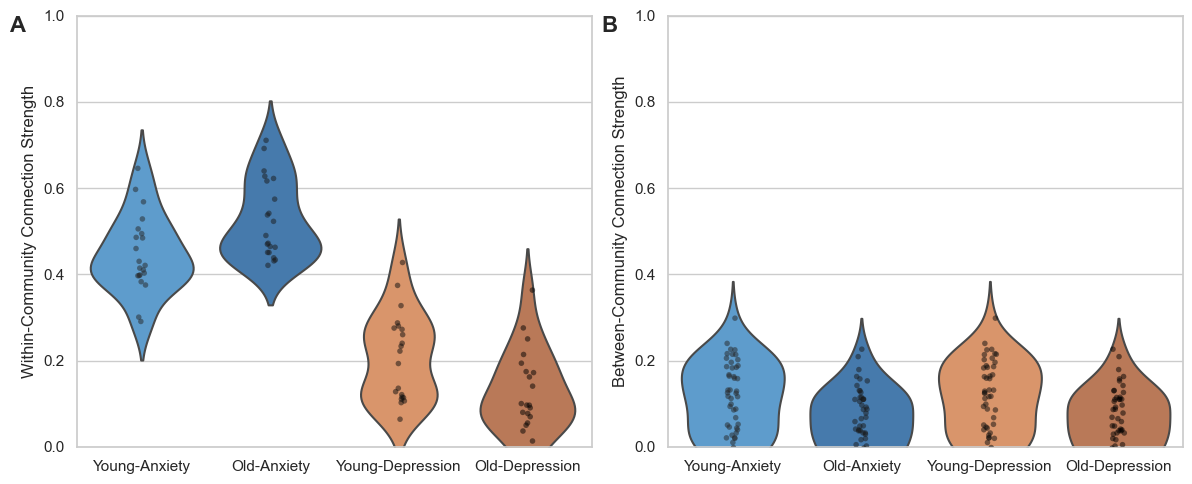


**Figure S2. Centrality metrics for the middle-aged group (ages 46–64 years).**(A) Strength centrality, representing the sum of connection weights for each symptom, is displayed for the middle-aged group. (B) Bridge strength centrality, which quantifies the importance of a symptom in connecting the depression and anxiety communities, is also shown for the same group. Nodes are colored in green and represent items from the Hospital Anxiety and Depression Scale (HADS), with anxiety symptoms (HA) and depressive symptoms (HD) ordered top to bottom.


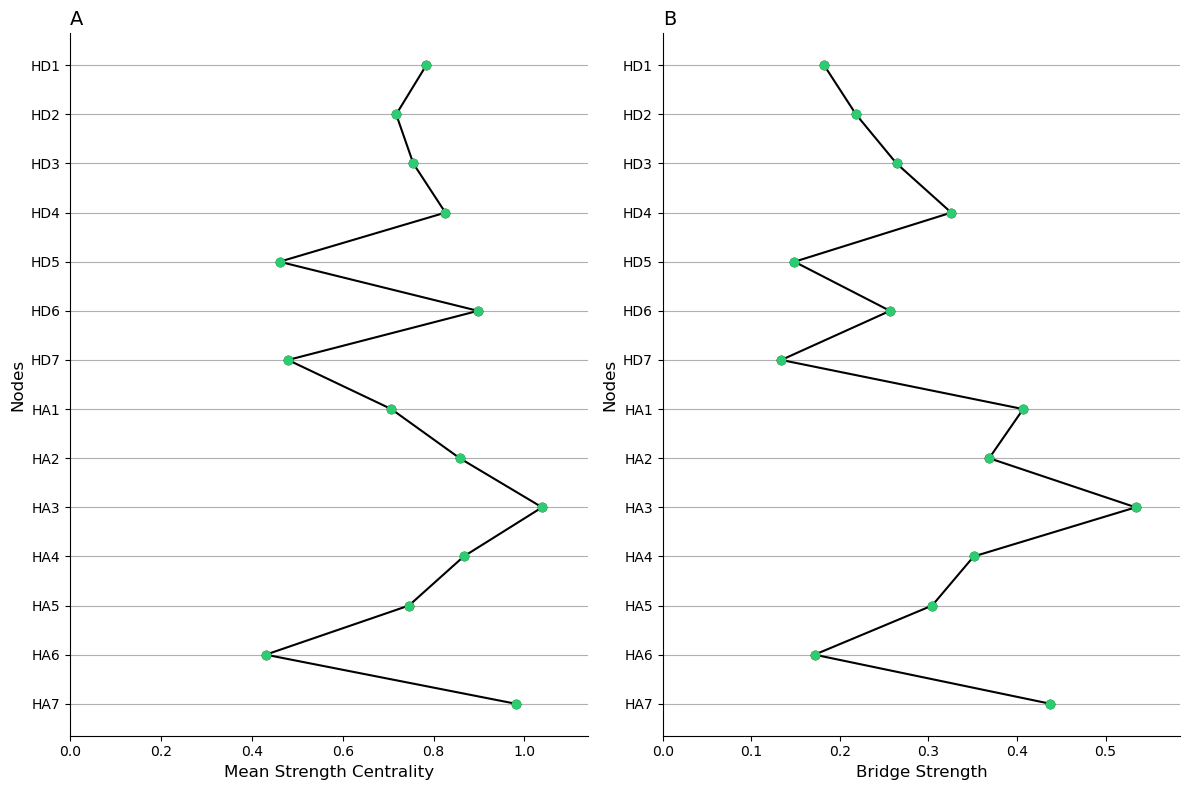


**Figure S3.** **Depression-anxiety symptom network in middle-aged adults (ages 46–64).**Network constructed using mutual information (MI) between HADS questionnaire items, with edges representing the magnitude of MI associations between items. Edge thickness and colour (as indicated in the colour bar) indicate normalized MI. Node colours represent communities detected by the Louvain algorithm (blue shade: anxiety community, orange shade: depression community).


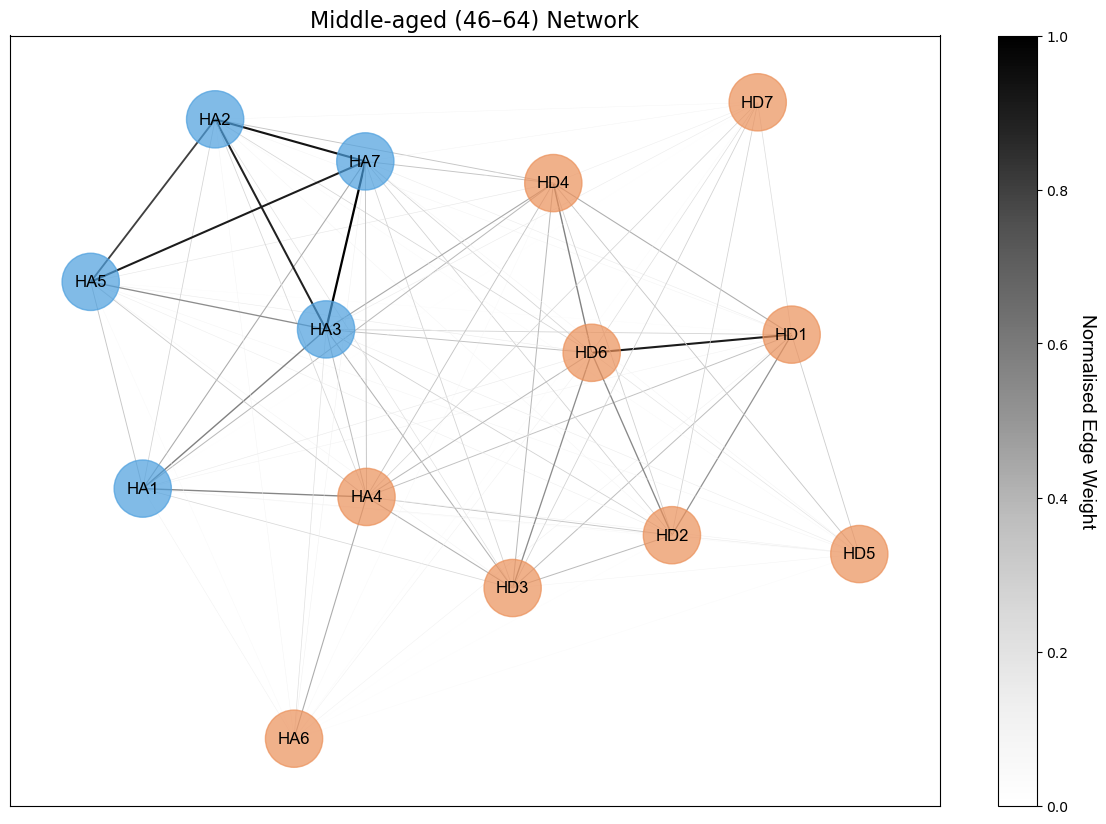


**References**

1. Shafto MA, Tyler LK, Dixon M *et al.* The Cambridge Centre for Ageing and Neuroscience (Cam-CAN) study protocol: a cross-sectional, lifespan, multidisciplinary examination of healthy cognitive ageing. *BMC neurology* 2014;**14**:1–25.

2. Bjelland I, Dahl AA, Haug TT *et al.* The validity of the Hospital Anxiety and Depression Scale: An updated literature review. *Journal of Psychosomatic Research* 2002;**52**:69–77.

3. Cuijpers P, Smit F. Subclinical depression: a clinically relevant condition? *Tijdschrift voor psychiatrie* 2008;**50**:519–28.

4. Szymkowicz SM, Woods AJ, Dotson VM *et al.* Associations between subclinical depressive symptoms and reduced brain volume in middle-aged to older adults. *Aging & Mental Health* 2019;**23**:819–30.

5. Meeks TW, Vahia IV, Lavretsky H *et al.* A tune in “a minor” can “b major”: a review of epidemiology, illness course, and public health implications of subthreshold depression in older adults. *J Affect Disord* 2011;**129**:126–42.

6. Rodríguez MR, Nuevo R, Chatterji S *et al.* Definitions and factors associated with subthreshold depressive conditions: a systematic review. *BMC Psychiatry* 2012;**12**:181.

7. Belvederi Murri M, Amore M, Respino M *et al.* The symptom network structure of depressive symptoms in late-life: Results from a European population study. *Mol Psychiatry* 2020;**25**:1447–56.

8. Djukanovic I, Carlsson J, Årestedt K. Is the Hospital Anxiety and Depression Scale (HADS) a valid measure in a general population 65–80 years old? A psychometric evaluation study. *Health and Quality of Life Outcomes* 2017;**15**:193.

9. Beard C, Millner AJ, Forgeard MJC *et al.* Network analysis of depression and anxiety symptom relationships in a psychiatric sample. *Psychological Medicine* 2016;**46**:3359–69.

10. Strauss GP, Esfahlani FZ, Galderisi S *et al.* Network Analysis Reveals the Latent Structure of Negative Symptoms in Schizophrenia. *Schizophrenia Bulletin* 2019;**45**:1033–41.

11. Wolpe N, Vituri A, Jones PB *et al.* The longitudinal structure of negative symptoms in treatment resistant schizophrenia. *Comprehensive Psychiatry* 2024;**128**:152440.

12. Borsboom D, Cramer AOJ. Network analysis: an integrative approach to the structure of psychopathology. *Annu Rev Clin Psychol* 2013;**9**:91–121.

13. Fruchterman TMJ, Reingold EM. Graph drawing by force‐directed placement. *Softw Pract Exp* 1991;**21**:1129–64.

14. Hagberg A, Swart PJ, Schult DA. *Exploring Network Structure, Dynamics, and Function Using NetworkX*. Los Alamos National Laboratory (LANL), Los Alamos, NM (United States), 2008.

15. Blondel VD, Guillaume J-L, Lambiotte R *et al.* Fast unfolding of communities in large networks. *Journal of statistical mechanics: theory and experiment* 2008;**2008**:P10008.

16. Bekhuis E, Schoevers RA, Van Borkulo CD *et al.* The network structure of major depressive disorder, generalized anxiety disorder and somatic symptomatology. *Psychological medicine* 2016;**46**:2989–98.

17. Fried EI, Epskamp S, Nesse RM *et al.* What are “good” depression symptoms? Comparing the centrality of DSM and non-DSM symptoms of depression in a network analysis. *Journal of Affective Disorders* 2016;**189**:314–20.

18. Park S-C, Kim D. The Centrality of Depression and Anxiety Symptoms in Major Depressive Disorder Determined Using a Network Analysis. *Journal of Affective Disorders* 2020;**271**:19–26.

19. Bringmann LF, Elmer T, Epskamp S *et al.* What do centrality measures measure in psychological networks? *Journal of Abnormal Psychology* 2019;**128**:892–903.

20. Jones PJ, Ma R, McNally RJ. Bridge Centrality: A Network Approach to Understanding Comorbidity. *Multivariate Behavioral Research* 2021;**56**:353–67.

21. De Winter JC, Gosling SD, Potter J. Comparing the Pearson and Spearman correlation coefficients across distributions and sample sizes: A tutorial using simulations and empirical data. *Psychological methods* 2016;**21**:273.
